# ﻿A new species of giant *Eunice* (Eunicidae, Polychaeta, Annelida) from the east coast of Australia

**DOI:** 10.3897/zookeys.1118.86448

**Published:** 2022-08-24

**Authors:** Joana Zanol, Pat Hutchings

**Affiliations:** 1 Departamento de Invertebrados, Museu Nacional, Universidade Federal do Rio de Janeiro, Av. Bartolomeu de Gusmão, 875, Rio de Janeiro, RJ 20941-160 Brazil Universidade Federal do Rio de Janeiro Rio de Janeiro Brazil; 2 Australian Museum Research Institute, Australian Museum, 1 William Street, 2010, New South Wales, Australia Australian Museum Research Institute, Australian Museum Sydney Australia; 3 Department of Biological Sciences, Macquarie University, North Ryde 2109, Australia Macquarie University North Ryde Australia

**Keywords:** Bobbit worm, *Euniceaphroditois*, Port Stephens, taxonomy

## Abstract

A new giant species is described from New South Wales, Australia. *Eunicedharastii***sp. nov.** differs from described Australian species and is most similar to *E.aphroditois* (Pallas, 1788), *E.flavopicta* Izuka, 1912, and *E.kinbergi* Ehlers, 1868. The unique combination of features that characterizes the new species is irregular articulated prostomial appendages; antennae reaching back beyond chaetiger 4; branchiae starting at chaetiger 10, initially button-shaped and distinctly longer than notopodial cirri where best developed; dorsal fleshy knobs on anterior chaetal lobes; notopodial cirri pendulous, abrupt tapering from inflated bases; bidentate compound falcigerous chaetae with both teeth directed laterally, distal tooth much shorter than proximal tooth in median and posterior chaetigers; and dark bidentate subacicular hooks starting at chaetiger 58, tapering to a small head with both teeth directed distally, and proximal tooth much larger than minute and spur-like distal tooth. This new species lives in sandy sediments in coastal waters 1–8 m deep. It is highly mobile and not easy to collect, which may explain why it was not described before.

## ﻿Introduction

*Eunice*, type genus of Eunicidae, is polyphyletic and currently characterized by plesiomorphic characters, such as the presence of three antennae, a pair of palps and a pair of peristomial cirri, the absence of regular articulations on prostomial appendages, presence of limbate, thin pectinate, compound bidentate falciger or spiniger chaetae, and dark uni- or bidentate subacicular hooks (Zanol and Budaeva 2021). The genus includes around 250 valid species (Read and Fauchald 2021). However, this number is likely to be an over-estimate because some species of the revalidated genera *Leodice* and *Nicidion* are still formally classified within *Eunice* (Zanol et al. 2021). Species of *Eunice* are present in all oceans in soft and hard substrates and various depths, but they are more common in biogenic hard substrates in shallow tropical waters (Fauchald 1992). The length of specimens of the genus is highly variable with longest species reaching a few meters in length (Fauchald 1992). These long species are considered giant due to their enormous length in comparison to the average length of *Eunice* species (e.g., Pruvot and Racovitza 1895; Salazar-Vallejo et al. 2011). Here, we consider giant species those with complete specimens reaching at least 1 m in length and 1 cm in maximum width.

Giant species are found in subtidal and intertidal zones of tropical and temperate oceans. The classification of these species has a long history of confusion. Many species have been synonymized with *Euniceaphroditois* (Pallas, 1788) (e.g., Fauvel 1932), leading to the common association of this name with a certain morph (large specimens bearing branchiae with many filaments, dark aciculae and dark subacicular hooks), and the underestimation of species diversity within the large-bodied morph (Fauchald 1992; Salazar-Vallejo et al. 2011). A more restricted and clear characterization of the species was made available by Fauchald (1992), who maintained only *Leodicegigantea* Lamarck, 1818 (no type specimen available, type locality La Réunion, Indian Ocean) as a junior synonym of *E.aphroditois* (no type specimen available, type locality Sri Lanka, Indian Ocean) and described two specimens from La Réunion (for a different opinion on this synonym see Salazar-Vallejo et al. 2011). In future, giant morphs identified as *E.aphroditois* should be re-examined and their true identity confirmed (e.g., Parapar and Harto 2001; Zanol and Bettoso 2006; Salazar-Vallejo et al. 2011; Schulze 2011).

Giant morphs from Indian and Pacific Oceans are almost always identified as *E.aphroditois* (e.g., Lachat and Haag-Wackernagel 2016), which is the only giant species recorded from Australian waters. *Euniceaphroditois* has been reported from East, South and West Australia in habitats such as sand, sponges, coral rubble, and under rocks from intertidal to subtidal (Zanol et al. 2020). Such diversity of habitats and widespread distribution is unexpected for eunicid species and detailed taxonomic re-analyses of widespread eunicid species have shown that multiple species have been confused as one (Iannotta et al. 2007; Lewis and Karageorgopoulos 2008; Glasby and Hutchings 2010; Schulze and Timm 2011; Molina-Acevedo and Carrera-Parra 2015; Zanol et al. 2016; Kara et al. 2020).

In this study, we describe a new species of giant *Eunice* from the subtidal zone of the east Australian coast (Southwest Pacific Ocean).

## ﻿Materials and methods

Specimens were collected during scuba dives at depths of 1–8 m by attracting an individual worm out of its tube through enticement with crushed pilchard, *Sardinopssagax* (Jenyns, 1842). Once the individual extended far enough out of the tube, it was quickly grabbed from behind the head and pulled out of its tube (see Suppl. material 1). Specific density was calculated during the scuba dive by a visual census of four transects, each 30 × 1 m.

Collected specimens were fixed and preserved in 95% ethanol. Preserved specimens were photographed with a Canon EOS 7D with a Macro EF 100 mm and the Spot Flex CCD 15.2 fitted on a Leica MZ16 Stereo microscope at the Australian Museum. The software Helicon Focus 5.3 was used for focus stacking. Parapodia (4, 15, 45,100, 200, 300, 400, 470) were removed from the holotype and paratype, dehydrated in ethanol, critically point dried, coated with 20 nm of gold, and examined under a JEOL JSM-6480 scanning electron microscope (SEM) at Macquarie University, Sydney. Additional parapodia (3, 80, 160, 240, 300, 380, 460) were removed, mounted in glycerine on a cavity slide and photographed using an Olympus DP 74 fitted on an Olympus Compound Microscope BX53, then the images were processed by Olympus cellSens software.

We describe the holotype and include values of paratype in parentheses. The general format of the description follows Fauchald (1992). Nomenclature used to describe articulation of prostomial appendages and cirri, and shape of chaetal lobe follows Zanol et al. (2014); shape of maxillae I and II follows Molina-Acevedo and Idris (2021); and shape of pectinate chaetae follows Carrera-Parra and Salazar-Vallejo (1998) and Zanol et al. (2016). Measurements of body length start at the anterior end of prostomium, they do not consider the length of prostomial appendages. All material is deposited in the Australian Museum, Sydney. The species is registered in ZooBank under urn:lsid:zoobank.org:act:63BC2367-9654-45DA-8021-FD17584DFFDC.

## ﻿Results

### ﻿Family Eunicidae

***Genus* Eunice**

#### ﻿ Eunice dharastii sp. nov.

Taxon classificationAnimaliaEunicidaEunicidae

BFC45050-4831-5B2D-9E7C-F99DC97D5DF1

https://zoobank.org/63BC2367-9654-45DA-8021-FD17584DFFDC

Figs 1 , 2

##### Material examined.

***Holotype*.** Australia • New South Wales, Nelson Bay, Port Stephens Main Beach; 32°42'54.91"S, 152°9'1.12"E; 8 m depth; Aug. 2012; D. Harasti leg.; AM W.53870. ***Paratype*.** Australia • 1 same data as for holotype; AM W.41747.

##### Comparative material.

Australia • 1 incomplete with 80 chaetigers, 120 mm in length and 20 mm maximum width Eunicecf.aphroditois; New South Wales, Nelson Bay, Port Stephens; AM W.140.

##### Description.

**Figure 1.**
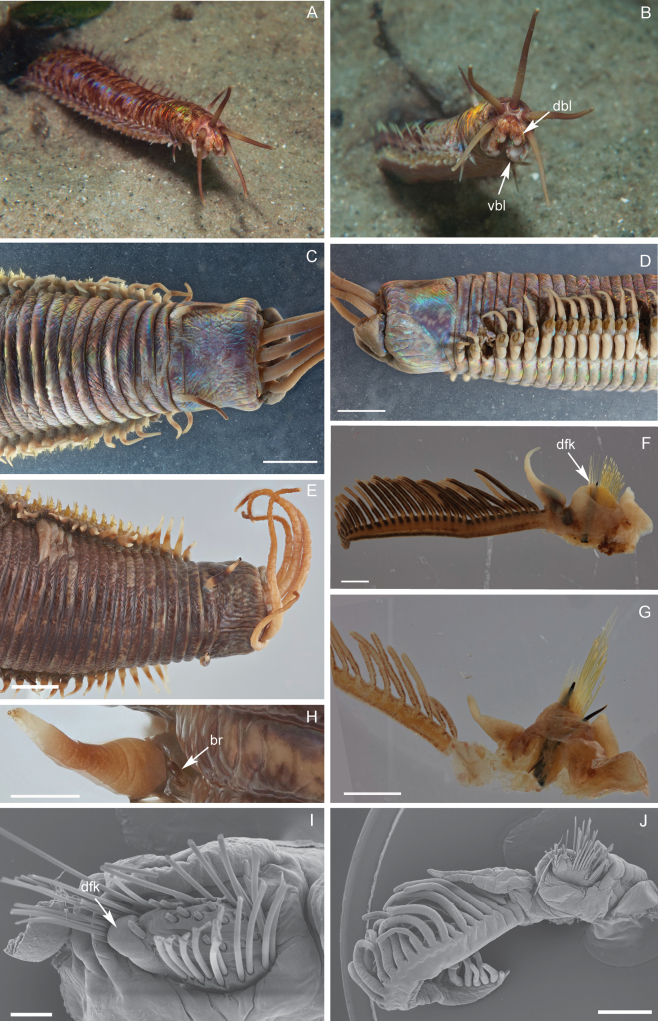
*Eunicedharastii* sp. nov. **A** anterior end of live specimen coming out of its burrow, dorsal view **B** anterior end of live specimen coming out of its burrow, anterior view **C** anterior end, dorsal view **D** anterior end, lateral **E** anterior end, dorsal view **F** parapodia, chaetiger 34, anterior view **G** parapodia from posterior chaetiger of the fragment, anterior view **H** branchiae and notopodial cirrus, chaetiger 10 **I** parapodia, chaetiger 4, upper view **J** parapodia, chaetiger 90, anterior view. br, branchiae; dbl, dorsal buccal lip; dfk, dorsal fleshy knob, vbl, ventral buccal lip. **I, J** scanning electron microscopy. **C, D** holotype AM W.53870 **E–J** paratype AM W.41747. Scale bars: 0.2 mm (**I**); 1 mm (**F, G, H, J**); 5 mm (**C, D, E**).

Live specimens: iridescent reddish with lighter patches on prostomium, peristomium, and along the body (Fig. 1A, B). Prostomium appendages, peristomial cirri, and notopodial cirri red to brown, uniformly colored, with lighter areas close to proximal half and at distal ends. Dorsal and ventral buccal lips whitish at distal end, standing out from posterior part of prostomium (Fig. 1B; see Suppl. material 2).

Fixed specimens iridescent brown to purple with lighter patches. Only peristomial cirri and few notopodial cirri retain color pattern of live specimens, prostomial appendages beige (Fig. 1A–C). Specimens are very curled and rigid because of the 95% ethanol fixation, making measurement of length and width difficult.

Holotype incomplete, with 520 chaetigers, in two pieces; first with 300 chaetigers, 200 well preserved + 100 slightly flaccid, and second with 220 chaetigers, all slightly flaccid; total length 980 mm; length through chaetiger 10 20 mm; width at chaetiger 10 without/with parapodia 12/15 mm, maximum width at chaetiger 18 without/with parapodia 18/22 mm, from chaetiger 18 width fairly uniform for following 200 chaetigers. Many parapodia with broken chaetae.

Paratype incomplete with 782 chaetigers in three pieces, first with 250 chaetigers, second with 222, all slightly flaccid, and third with 310 chaetigers; total length 1170 mm; length through chaetiger 10 20 mm; width at chaetiger 10 without/with parapodia 16/19 mm; maximum width at chaetiger 100 without/with parapodia 18/23 mm. Body almost semicircular anteriorly, becoming more flattened around chaetiger 70–80.

Prostomium with dorsal buccal lips as paired median dorsal ridges, obliquely truncate, with thickened lateral margins and median sulcus narrow (Fig. 1B). Dark eyes present outside lateral antennae. Median, lateral antennae and palps reaching back, respectively, to chaetigers 4 (9), 5 (9) and at least until middle of first peristomium ring (3) (antennae and palps are very rigid and difficult to manipulate in holotype; measurements are estimates). Prostomial appendages not evenly spaced, palps isolated by a small gap from lateral antennae; arranged in semicircle, palps partially in front of lateral antennae (Fig. 1B). Ceratophores of median and lateral antennae and palpophores short and ring-shaped. Ceratostyles of median and lateral antennae and palpostyles irregularly articulated; tapering (Fig. 1E). Peristomium cylindrical; separation between first and second rings only visible on dorsal and ventral sides; ventrally second ring much shorter than dorsally (Fig. 1C–E). Dorsally first ring 5/6 of total length of peristomium. Ventrolateral lips muscular and inflated (Fig. 1D). Peristomial cirri reaching a little more anterior than middle of first peristomial ring; irregularly articulated; tapering (Fig. 1C, E).

**Figure 2.**
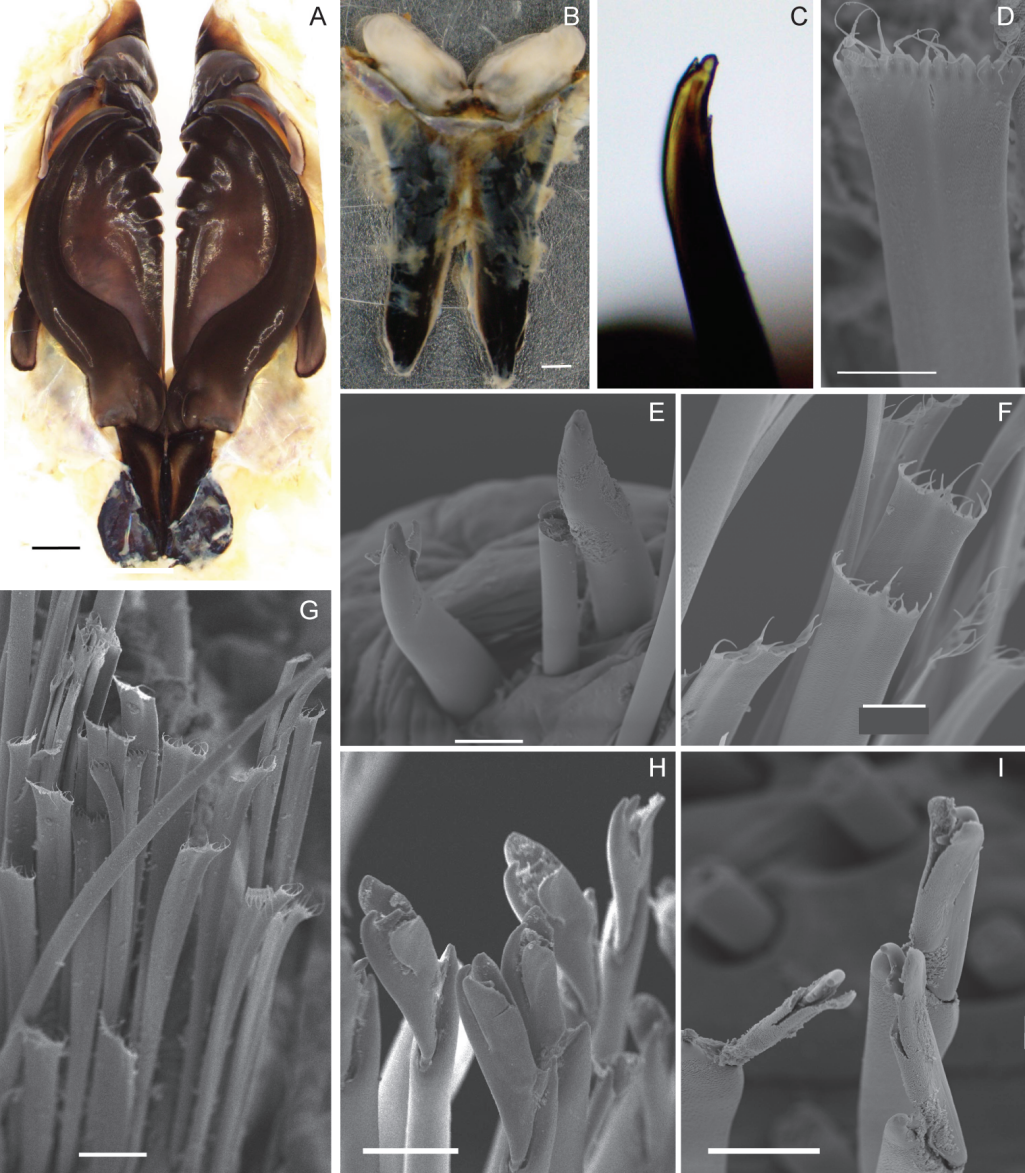
*Eunicedharastii* sp. nov. (paratype AM W.41747) **A** maxillae, dorsal view **B** mandible, ventral view **C** subacicular hook from posterior chaetiger of the fragment **D** pectinate chaeta, chaetiger 200 **E** subacicular hook, chaetiger 300 **F** pectinate chaetae, chaetiger 300 **G** pectinate chaetae, chaetiger 240 **H** falciger chaetae, chaetiger 300 **I** falciger chaetae, chaetiger 4. Scale bars: 20 μm (**D, F**); 50 μm (**C, E, G, H, I**); 1 mm (**A, B**).

Maxillary formula 1+1, 7+7, 7+0, 4+7, 1+1, 1+1 (Fig. 2A). Carrier with lateral anterior sclerotized margins almost parallel to each other, abrupt tapering after initial 1/3 of its length. MxI about 2.5 times longer than carrier, lacking a curvature at internal basal edge, with a curvature at outer basal edge, falcal arch extended. MxII with teeth distributed along more than half its length, posterior end wide with distinct thickened outer ridge. MxIII short; part of distal arc with left MxIV and V. MxVI ridge like with a narrow distal tooth. Mandibles calcareous cutting plates with ellipsoid shape (Fig. 2B).

Branchiae present from 10 (10) until at least chaetiger 520, end of branchiae not recorded (branchiae ends well before pygidium on chaetiger 492); first with just 1 button-shaped filament around 1/5 of dorsal cirri length (Fig. 1H), around chaetiger 22 as long as notopodial cirri, number of filaments rapidly increasing to 38 (26); best developed branchiae from about chaetiger 40 through subsequent chaetigers with thick tapering stems, around 2.5 to 4 times longer than longest filament and notopodial cirri (Fig. 1F, J); becoming shorter at end of most posterior fragment (becoming shorter at end of distribution).

Chaetal lobes truncate along whole fragment, posterior increasingly oblique; anterior with dorsal fleshy knob and neuroaciculae emerging posterior to it (Fig. 1F, I); neuroaciculae near dorsal edge in all parapodia. Prechaetal lobe low transverse fold until end of fragment. Postchaetal lobes round/truncate, longer than chaetal lobe at anterior end (Fig. 1D), decreasing along the body, low transverse fold shorter than chaetal lobe by end of fragment. Anteriormost ventral cirri thumb-shaped to tapering, becoming basally inflated from about chaetiger 4 or 5; inflated bases elongate, ridge-like decreasing towards posterior end; free tip round to slightly tapering in all chaetigers, clearly separated from base (Fig. 1D, F). Notopodial cirri pendulous, abrupt tapering from inflated bases, irregularly articulated (Fig. 1D–F, H).

Slender, tapering limbate chaetae longer than all other chaetae present in all chaetigers. Pectinate chaetae thin anodont with flattened shafts; tapering smoothly subdistally or near proximal end along whole fragment (Fig. 2D, F, G). Numbers of teeth variable, 10–14 (*N* = 21, mode = 11); each tooth flattened, distally tapering abruptly to slender hair-like tip; all with similar lengths. Distal ends of compound falciger chaetae shafts a little wider than proximal ends along whole fragment. Appendages of compound falciger chaetae with variable lengths within a chaetal bundle of anterior parapodia, longest in anterior parapodia; shortest appendages of anterior chaetigers as long as appendages in median and posterior chaetigers, all with similar lengths; bidentate with both teeth directed laterally; both teeth about same length in anterior chaetigers, distal tooth much shorter than proximal tooth in median and posterior chaetigers; guards asymmetrically blunt (Fig. 2H, I). Neuroaciculae distinctly dark along all its length, double in most parapodia, some posterior parapodia with single acicula; tapering to blunt or sharp tips (Fig. 1F, G). Subacicular hooks present from chaetiger 58 (53); initially one per parapodium, increasing to two, reaching a maximum of four at chaetiger 81 (85) subsequent parapodia with three or four, most posterior with two; distinct, dark along all its length, with distinct dark core and clear sheath at distal end; bidentate tapering to small head, distal tooth minute, spur-like, proximal tooth much larger, both teeth directed distally (Fig. 2C, E). In both types many chaetae broken.

Posterior end of body and pygidium missing.

##### Habitat and specific density.

Water depth, 1–8 m, in tubes in coarse sand substrates; also occurs in sandy habitats to the west and east of the type locality in same depth range. Average specific density in Nelson Bay main beach 3.5 ± 0.6 individuals per 30 m^2^.

##### Type locality.

Nelson Bay Main Beach (32°42'54.91"S, 152°9'1.12"E), Port Stephens, New South Wales, Australia.

##### Etymology.

The species is named in honor of Dr David Harasti, who collected the specimens, donated them to the Australian Museum, and first suspected they were a species new to science.

##### Remarks.

*Eunicedharastii* sp. nov. is most similar to *E.aphroditois*, *E.flavopicta* Izuka, 1912 and *E.kinbergi* Ehlers, 1868 in having the prostomium with dorsal buccal lips as paired median dorsal ridges; MxVI present; branchiae longer than notopodial cirri, with stem much longer and thicker than filaments; pectinate chaetae thin anodonts with flattened shafts tapering smoothly subdistally or near proximal end; bidentate compound falcigers, dark paired tapering/blunt neuroaciculae and dark bidentate subacicular hooks (Izuka 1912; Fauchald 1992). The unique combination of features that characterizes *Eunicedharastii* sp. nov. and differentiate it from these three species are: irregular articulated prostomial appendages; antennae reaching back beyond chaetiger 4; branchiae starting at chaetiger 10, initially button-shaped; best developed branchiae distinctly longer than notopodial cirri; dorsal fleshy knob on anterior chaetal lobe; notopodial cirri pendulous (sensu Fauchald 1992), abrupt tapering from inflated bases; bidentate compound falciger chaetae with both teeth directed laterally, distal tooth much shorter than proximal tooth in median and posterior chaetigers; and dark bidentate subacicular hooks starting at chaetiger 58, tapering to small head with both teeth directed distally, proximal tooth much larger than minute and spur-like distal tooth (Table 1). The unusual shape of the subacicular teeth is shared only with *E.aphroditois*, *E.borneensis* Grube, 1878 (type locality North Borneo), and *E.mutabilis* Gravier, 1900 (type locality Djibouti; Fauchald 1992). The latter two are clearly distinct from *E.dharastii* sp. nov. in several features, such as the absence of MxVI, shape of branchiae and chaetae.

**Table 1.** Main morphological features that differentiate *Eunicedharastii* sp. nov. from the closest species.

	*Eunicedharastii* sp. nov.	*Euniceaphroditois* (Pallas, 1788)	*Euniceflavopicta* Izuka, 1912	*Eunicekinbergi* Ehlers, 1868
Data source	Present description	Fauchald 1992	Izuka 1912; Fauchald 1992	Fauchald 1992
Type locality	Australia, New South Wales Nelson Bay, Port Stephens Main Beach	Sri Lanka	Japan^1^	South Africa, Cape of Good Hope
Irregular articulation of prostomial appendages (antennae and palps)	Present	Absent	Present	Present
Region median and lateral antennae reach when folded back	holotype- chaetiger 4 (M), 5 (L)paratype- chaetiger 9 (M), 9 (L)	posterior end of peristomium (M, L)	chaetiger 2 (M), chaetiger 1 (L)^2^	chaetiger 1 or 4 (M), chaetiger 1 (L)
Branchiae present from chaetiger	10	6	5^2^	8–9
Length of best developed branchiae in relation to notopodial cirri	Distinctly longer	Distinctly longer	Distinctly longer	About as long as
Dorsal fleshy knob on anterior chaetal lobe	Present	Absent	Absent	Absent
Shape of notopodial cirri	Pendulous (sensu Fauchald 1992); abrupt tapering from inflated bases	Smooth tapering from inflated bases	Pendulous (sensu Fauchald 1992); smooth tapering from with inflated bases	Smooth tapering from slightly inflated bases
Direction of teeth of compound chaetae	Both directed laterally	Not described	Proximal directed laterally, distal directed distally	Both directed laterally
Relative length of distal and proximal teeth of compound chaetae	Anterior both similar in length, median and posterior distal tooth much shorted than proximal tooth	Not described	Distal tooth shorter than proximal tooth^3^, variation not described	Both similar in length or distal tooth longer than proximal tooth
Shape of the distal end and direction of teeth of the subacicular hook	Tapering to small head with both teeth directed distally	Tapering to small head with both teeth directed distally	Head bent with both teeth directed laterally	Distinct head with proximal tooth directed laterally, distal tooth directed distally
Shape and relative size of proximal and distal teeth of subacicular hook	Proximal tooth distally blunt, distal tooth minute spur-like, much smaller than proximal tooth	Both teeth distally blunt, distal tooth smaller than proximal tooth	Both teeth triangular, distal tooth much smaller than proximal tooth	Both teeth triangular, distal tooth smaller than proximal tooth
Subacicular hook present from chaetiger, distribution	53 or 58, uniform present in all chaetigers thereafter	200, scattered missing in several chaetigers thereafter	Not described	123, scattered missing in several chaetigers thereafter

^1^The original description contains two localities in Japan: Misaki in Sagami Province (currently Kanagawa Prefecture) and Ushibuka in Higo Province (currently Kumamoto Prefecture) (Izuka 1912). ^2^Numbers differ from those in Izuka (1912), because he considered peristomial rings as two segments. ^3^Izuka (1912) described denticles between proximal and distal teeth. In the illustration (Plate XIV, fig. 4 in Izuka 1912), these denticles appear to be the distal end of the guards. The presence of these denticles needs to be revised. M, median antenna. L, lateral antennae.

The examined specimen from Port Stephens identified as E.cf.aphroditois (Zanol et al. 2020) differs from the new species in the length of the prostomial appendages, median and lateral antennae folding back to chaetiger 1 and palps to the first peristomial ring; and the start of branchiae at chaetiger 11 with five short filaments. Thus, it is here considered a different species. At least three *Eunice* species live in sympatry in the vicinity of the type locality (Port Stephens), E.cf.aphroditois, *E.impexa* Grube, 1878 (Zanol et al. 2020), and *E.dharastii* sp. nov. Besides *E.aphroditois*, *E.confusus* Zanol, Hutchings & Fauchald, 2020 is the most similar species to *E.dharastii* sp. nov. reported from Australian waters. Nevertheless, they only share a similar shape of the prostomium, peristomium and peristomial cirri, the prostomial appendages are irregularly articulated, maxillary formula (but not shape of plates and carrier), shape of anterior pectinate chaetae and aciculae. Considering previous knowledge on the diversity of morphological features, the observed differences are enough to consider the analyzed specimens a new species to science. Molecular data for this species will be available in a subsequent paper.

## ﻿Discussion

Here we describe a new *Eunice* species to science, which is at least 1 m long. Despite the large size and the shallow water habitat of some giant *Eunice* species, their diversity is not fully understood. This is due to the wide synonymizing of species, poor understanding of their biology and morphological variation, their concealed habitats, and difficulty in sampling (Fauchald 1992; Parapar and Harto 2001; Salazar-Vallejo et al. 2011; Schulze 2011; Escobar-Ortega et al. 2022).

Specimens from Australia identified and described as *E.aphroditois* (Ehlers 1868; McIntosh 1885; Augener 1913; Fauvel 1917; Monro 1931) assume a high intraspecific diversity and may in fact represent more than one species (Fauchald 1992; Salazar-Vallejo et al. 2011). Despite the diversity found in these descriptions, they all contrast from *E.dharastii* sp. nov. in the shape of the chaetae and, in some cases, in the start of branchiae (e.g., McIntosh 1885). However, we cannot rule out that at least some of the previous records of *E.aphroditois* from Australian waters are in fact *E.dharastii* sp. nov. and that online photographs of the species may have been available prior to this description (e.g., Salazar-Vallejo et al. 2011). The name *E.aphroditois* may, therefore, have obscured other undescribed species.

However, the large variation in size may lead to the identification of one species as belonging to several species. Giant species are described from large specimens, but which were once small during earlier life stages (Pruvot and Racovitza 1895; Schulze 2011). Thus, the identification of giant specimens should also include comparisons with species described from smaller specimens bearing in mind that some features vary with size. Subacicular hooks tend to appear for the first time in more posterior chaetigers and to be irregularly distributed in giant species (Fauchald 1992), which is probably due to a progressive loss with size increase (Miura 1977). Maximum number of branchial filaments tend to increase with size (Parapar and Harto 2001). On the other hand, the shape of prostomium and dorsal buccal lips, branchiae with more than 10 filaments where best developed, a type A pattern of branchial distribution (sensu Miura 1986), the dark color of aciculae and subacicular hooks all appear to be useful in recognizing similar species within this group of *Eunice* despite their size. By contrast, for the identification of species, it appears to be more informative to consider the relative length of prostomial appendages, morphology of the jaws, shape of ventral cirri, start of branchiae, shape and relative size (length and width) of branchial stem and filaments, shape and relative size of notopodial cirri, and shape and variation along the body of compound falciger chaetae, pectinate chaetae, and teeth of bidentate subacicular hooks, fewer features than those suggested by Pruvot and Racovitza (1895). The consideration of the shape of pectinate chaetae and its variation along the body in the diagnosis of species of the eunicid genera such as *Marphysa* have improved the identification and differentiation of species (e.g., Martin et al. 2020).

Despite the large size of giant species, they are concealed in deep burrows from which the anterior end emerges for feeding. Other than by dredging, regular substrate sampling is unlikely to sample them. They can rapidly retreat back into their extensive burrow when sensing any vibration. Many of the reports of these large species come from dredged samples (e.g., McIntosh 1885), washed up specimens (e.g., Pruvot and Racovitza 1895), or hand-collected specimens (not an easy task; Escobar-Ortega et al. 2022), usually, from places with high numbers of scuba divers or where giant species are used as bait (e.g., Bettoso et al. 1998). Thus, we can expect that the diversity of giant species is underestimated and that even places where the *Eunice* fauna is relatively well studied hide new giant species. Knowledge of these large species will aid to improve our understanding of their diversity, morphological variation, and evolution of body size in Eunicidae.

## Supplementary Material

XML Treatment for Eunice dharastii

## Supplementary materials

Supplementary material 1 Video S1 Data type Video file. Explanation note Attempt to catch a specimen of *E.dharastii* sp. nov. https://binary.pensoft.net/file/734652This dataset is made available under the Open Database License (http://opendatacommons.org/licenses/odbl/1.0/). The Open Database License (ODbL) is a license agreement intended to allow users to freely share, modify, and use this Dataset while maintaining this same freedom for others, provided that the original source and author(s) are credited.Author: David Harastii

Supplementary material 2 Video S2 Data type Video file. Explanation note Anterior end of *E.dharastii* sp. nov. live specimen coming out of burrow. https://binary.pensoft.net/file/734653This dataset is made available under the Open Database License (http://opendatacommons.org/licenses/odbl/1.0/). The Open Database License (ODbL) is a license agreement intended to allow users to freely share, modify, and use this Dataset while maintaining this same freedom for others, provided that the original source and author(s) are credited.Author: David Harastii
